# Development of the ocellar visual system in *Drosophila melanogaster*

**DOI:** 10.1111/febs.16468

**Published:** 2022-05-11

**Authors:** Claude Bernard Jean‐Guillaume, Justin P. Kumar

**Affiliations:** ^1^ Department of Biology Indiana University Bloomington IN USA

**Keywords:** compound eye, *Drosophila*, Hofbauer‐Buchner eyelet, ocelli

## Abstract

The adult visual system of the fruit fly, *Drosophila melanogaster*, contains seven eyes—two compound eyes, a pair of Hofbauer‐Buchner eyelets, and three ocelli. Each of these eye types has a specialized and essential role to play in visual and/or circadian behavior. As such, understanding how each is specified, patterned, and wired is of primary importance to vision biologists. Since the fruit fly is amenable to manipulation by an enormous array of genetic and molecular tools, its development is one of the best and most studied model systems. After more than a century of experimental investigations, our understanding of how each eye type is specified and patterned is grossly uneven. The compound eye has been the subject of several thousand studies; thus, our knowledge of its development is the deepest. By comparison, very little is known about the specification and patterning of the other two visual systems. In this Viewpoint article, we will describe what is known about the function and development of the *Drosophila* ocelli.

## Graphical abstract

The ocelli are visual organs that, along with the compound eyes and Hofbauer‐Buchner eyelets, are responsible for a variety of visual and circadian behaviors. In the fruit fly, *Drosophila melanogaster*, a number of loss‐of‐function mutants have been identified that negatively impact the specification, number, growth, spacing, and positioning of the ocelli. These mutants provide key insights into the gene regulatory networks that control the development of these important visual organs.

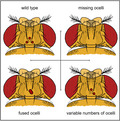

**Abbreviations**
ara: araucan
ato: atonal
caup: caupolican
ci: cubitus interruptus
dve: defective proventriculus
Egfr: epidermal growth factor receptor
en: engrailed
ey: eyeless
eya: eyes absent
eyg: Eyegone
gl: glass
GRN: gene regulatory network
hh: hedgehog
iOC: inter‐ocellar cuticle
mirr: mirror
N: Notch
norpA: no receptor potential A
oc: ocelliless
otd: orthodenticle
pnt: pointed
ptc: patched
R: photoreceptor
rdgB: retinal degeneration B
Rh: rhodopsin
sev: sevenless
so: sine oculis
toy: twin of eyeless
trp: transient receptor potential
vn: vein
wg: wingless

## Introduction

The adult fruit fly, *Drosophila melanogaster*, has seven eyes: a pair of compound eyes, a trio of ocelli, and two extra‐retinal eyelets [1]. Together, these three systems are responsible for entrainment of the light‐responsive circadian clock and for all visual behaviors that the fly needs to execute for proper feeding, mate selection, avoidance of predators, and flight navigation. The unique visual and circadian behaviors of each system are made possible by the distinctive physical structure of each eye and the downstream neural wiring patterns. As such, it is important to understand how each system is first specified and then patterned. Although an abundance of information on the structure and physiology of all three visual systems exists, detailed information on the development of these systems is primarily confined to the compound eye. Here, we will provide an overview of each visual system with particular emphasis on the ocellar system of *Drosophila*.

## The compound eyes

**Fig. 1:**
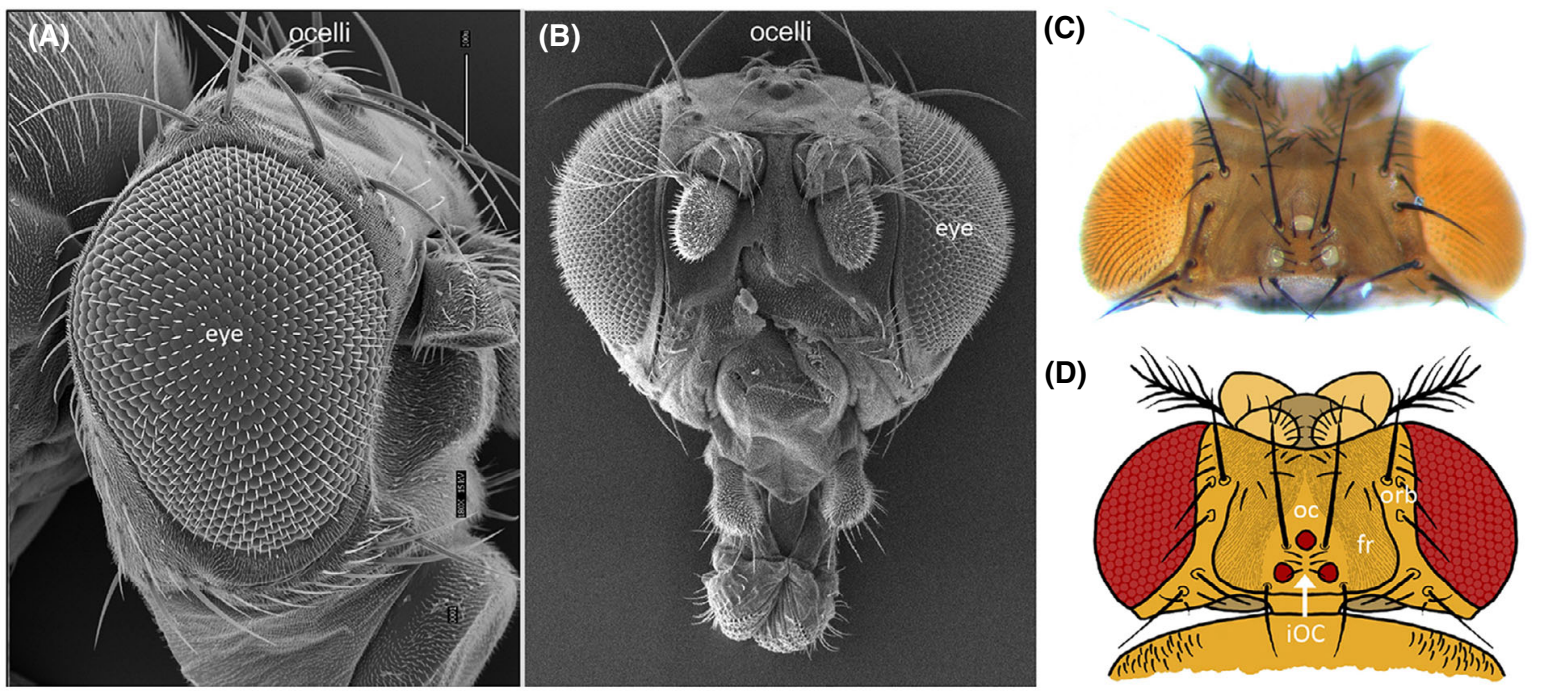
Organization of the adult Drosophila visual systems. (A, B) Scanning electron views of either side or head‐on views of the *Drosophila* head. The compound eyes occupy positions on the lateral sides of the fly head. The ocelli, by comparison, are located within the dorsal head vertex that lies between the two compound eyes. (C) Light microscope image of dorsal view of the adult head showing the three ocelli. (D) A schematic drawing of the adult head showing the different domains of the head vertex. oc = ocellar domain, fr = frons, and orb = orbital domain. Please note that the ocellar domain contains the three ocelli and the inter‐ocellar cuticle (iOC) that lies between the ocelli themselves.

The compound eyes of *Drosophila* are located on the lateral sides of the adult head and are each composed of approximately 750 unit eyes called ommatidia (Fig. 1A,B) [2]. Each unit eye contains eight photoreceptors (R1‐R8) and twelve non‐neuronal cone and pigment cells. These cells occupy stereotyped positions within the ommatidium and perform specialized functions. The photoreceptors convert light into electric signals, the cone cells secrete the overlying lens, and the pigment cells optically insulate each unit eye from its adjacent neighbors. Each photoreceptor neuron will express one of five different rhodopsin genes. The outer photoreceptors R1‐6 all express the Rh1 blue‐green‐sensitive opsin [3, 4]. The inner R7 neuron will express one of two ultraviolet‐sensitive opsins (Rh3/Rh4) [5, 6, 7]. And, the other inner photoreceptor (R8), which lies beneath the R7, will either express the Rh5 blue‐sensitive or the green‐sensitive Rh6 opsin [8, 9, 10]. This combination of rhodopsin proteins allows the flies to sense polarized light, motion, and see in color.

The compound eyes are derived from a pair of sac‐like larval structures called eye‐antennal disks [11, 12, 13, 14]. Overt patterning of the eye begins at the start of the third larval instar when a wave of differentiation initiates from the posterior margin of the disk. The leading edge of this differentiating wave can be visualized by a dorso‐ventral groove in the epithelium called the morphogenetic furrow. Over the course of three days, the furrow traverses across the retinal primordium and transforms the field of undifferentiated cells into an ordered array of unit eyes [2]. Within the ommatidium, cells adopt their fate in a stepwise manner akin to an assembly line. In short, the photoreceptors are specified first followed by the cone and pigment cells. The mechanosensory bristle complexes are added to the ommatidium last [2, 15, 16].

The compound eyes are responsible for phototactic movement, motion detection, pattern recognition, and color vision [17, 18, 19, 20, 21, 22, 23, 24, 25, 26, 27]. How well a compound eye carries out each of these behaviors is dependent upon several features that include the overall number of ommatidia, the physical dimensions of each unit eye, the number of photoreceptors per unit eye, the ratio of photoreceptors cells to second‐order neurons, the internal structure of the rhabdom (fused or open), the neural wiring of the unit eye (apposition, superposition, or neural superposition), the number of connections within the neural circuit, as well as the type and spectral properties of the opsin proteins. In addition to mediating various visual behaviors, the compound eye also contributes to the entrainment of the molecular circadian clock [1, 28, 29, 30, 31, 32].

## The Hofbauer‐Buchner eyelet

The extra‐retinal eyelets were discovered decades after the first major monographs on insect visual system structure and function predicted their existence [1, 17, 18]. The two eyelets participate, along with the compound eyes, in the entrainment of the molecular clock, and each one lies between one of the compound eyes and its associated optic ganglion [1, 29, 31]. The pair of four‐celled eyelets are derived from two bundles of larval photoreceptors that are called Bolwig organs—named after their discoverer, Niels Bolwig [33]. These comprise the larval visual system and along with class IV multidentric (md) neurons allow for complex phototactic behaviors. For example, juvenile larvae use these two sensory systems to detect light and then move away from it (negative phototaxis). Older larvae, by comparison, will engage in positive phototaxis by using these same organs/neurons to crawl toward the light [34, 35].

Each larval Bolwig organ contains a mixture of four blue‐sensitive (Rh5 expressing) and eight green‐sensitive (Rh6 expressing) photoreceptor neurons [29, 36, 37]. During pupal development, all of the original green‐sensitive photoreceptors are pruned away by programmed cell death. The remaining blue‐sensitive photoreceptors then completely change their spectral sensitivity by first terminating transcription of Rh5 and then by activating Rh6 expression. As such, each adult eyelet is comprised of just four Rh6 expressing green‐sensitive photoreceptors [29, 31, 38]. This change in rhodopsin expression represents a unique example of sensory plasticity in terminally differentiated neurons.

## Structure and function of the ocellar visual system in *Drosophila*

Ever since the ocelli were first described almost 300 years ago [39], anatomists and entomologists have carefully documented which insects have ocelli and which ones do not. Although there are exceptions, the general rule of thumb holds that ocelli are present within flying insects but not within ones that are grounded. Exceptions to this rule include some species of butterflies that lack ocelli altogether and a few species of termites, desert ants, and beetles that cannot fly but have ocelli [40, 41, 42, 43, 44, 45]. It appears that the ocelli have been lost or gained several times during the evolution of insects. The ocelli share several basic features with the ommatidia of the compound eye in that they contain photoreceptor neurons, lens‐secreting cone cells, and optically insulating pigment cells.

In *Drosophila*, the three ocelli are located between the compound eyes on the vertex of the adult head in a triangle pattern (Fig. 1C,D). Like the compound eyes, the ocelli are derived from the pair of larval eye‐antennal disks [13]. Each disk produces one of the two lateral (also called posterior) ocelli and one half of the medial (also called anterior) ocellus [46, 47]. During pupal development, the two halves of the medial ocellus are fused to each other when the two eye‐antennal disks are stitched together to make a single intact head covering [48, 49, 50]. Each adult ocellus consists of approximately 80 photoreceptors, all of which express the Rh2 violet‐sensitive rhodopsin [51, 52, 53], as well as a set of lens‐secreting cone cells and optically insulating pigment cells. The photoreceptor axons directly innervate the optic lobe, which is the part of the fly brain that is responsible for processing visual information. The optic lobe is comprised of four structural components: the lamina, the medulla, the lobula, and the lobula plate. Histological preparations demonstrated that ocellar photoreceptors directly innervate the latter two structures [22]. The lobula and lobula plate also receive information from the compound eye through intermediate connects that are relayed from the lamina and medulla [54]. This wiring pattern suggests that visual information received by the compound eye and ocelli is integrated within the deepest layers of the optic lobe and then passed on to the central brain complex [55].

Electrophysiological recordings of the *Drosophila* ocellus can be found in just a single paper [56]. In this study, it was shown that some but not all members of the phototransduction machinery are shared by compound eye and ocellar photoreceptors. The *transient receptor potential* (*trp*) and *retinal degeneration B* (*rdgB*) genes play important roles in the phototransduction response of compound eye photoreceptors [57, 58]. However, when the ocellar light response from these mutants was recorded, defects in phototransduction were detected in *trp*, but not *rdgB*, mutants. Behavioral studies further demonstrated that *no receptor potential A* (*norpA*), which is an essential component of the phototransduction cascade in compound eye photoreceptors, is completely dispensable for visual behaviors mediated by the ocelli [59].

The study by Labhart also showed that the ocellar photoreceptors are not divided into the same neuronal subtypes as within the ommatidium of the compound eye. For example, in the retina, mutations in the *sevenless* (*sev*) gene lead to the transformation of the R7 neuron into a cone cell [60, 61]. As a consequence, the eye is rendered insensitive to ultraviolet light [58, 62]. However, ocellar recordings from *sev* mutants are indistinguishable from wild type, suggesting that an R7 subtype does not exist within the ocelli. In the compound eye, several other neuronal subtypes exist (R8, R2/R5, R3/R4, and R1/6). A large library of transcription factors that specify the fate of each subtype has been identified by expression patterns and mutant analysis [63, 64, 65]. It would be interesting to see which of these transcription factors are expressed within the ocelli and if any of the neuronal subtypes that are present in ommatidium are also present in the ocellus.

Behavioral studies of *Drosophila* indicate that the ocelli contribute to a wide range of behaviors including sensing the horizon, flight stabilization, entrainment of the circadian clock, color choice, and phototaxis [59, 66, 67, 68, 69, 70, 71]. These behaviors are normally dominated by the compound eyes, and the ocelli appear to simply augment the visual response. In other words, flies can still execute these behaviors even when the ocelli are manually occluded with paint or genetically ablated. For example, flies with occluded ocelli have reduced (but not eliminated) phototactic responses [72]. Similarly, flies in which the ocelli have been genetically eliminated have reduced but still robust locomotor activity. They can also discriminate between different colors albeit not as efficiently as wild‐type flies [66]. Very few electrophysiology studies on the ocelli have been conducted in *Drosophila;* thus, there is still a lot to be learned about the physiological contributions of the ocelli to fly vision.

## Structure and function of the ocelli in non‐*Drosophila* species

In contrast to the compound eyes, the ocelli, in all species examined so far, have very poor resolving power and are, therefore, not particularly useful for pattern recognition. This is due to the fact that the ocellar plane of focus lies behind that of the retina and this results in an under‐focused image [73, 74, 75, 76]. Also, the slightly oval shape of each ocellus produces an astigmatism, which further degrades an already hazy picture [77]. As such, the image that the ocelli contribute to the insect brain is quite blurry. So, what is the role of the ocelli in visual behavior if it not to help the fly see clearly?

Interestingly, unlike the compound eyes, a universal set of visual behaviors cannot be attributed to the ocelli. This is in part because the number, location, internal structure, and neural circuitry of the ocelli differ from one species to another. For example, while *Drosophila* has three ocelli, the cockroach and most butterflies have just two, whereas some species of jumping bristletails can have up to sixteen ocelli [41, 78, 79, 80, 81, 82]. Contrastingly, while all three ocelli form a tight triangular pattern in *Drosophila*, they are well separated from each other in the locust [79, 83]. Additionally, while in an overwhelming number of species the ocelli are located on the external surface of the head, in some insects such as the sphinx moths the ocelli are positioned internally underneath the head epidermis [84]. These factors all impact the ability of the insect to stabilize itself while in flight and/or see in differing light conditions.

The function of the ocelli is also affected by variations in the overall size of the ocellus as well as the number of photoreceptors that are contained within each one. For example, the ocelli of nocturnal bees and ants are larger than those of their diurnal cousins and this, in part, allows them to forage, navigate, and orient themselves using celestial light that is 100 million times dimmer than daylight [85, 86]. The number of photoreceptors within each ocellus can also influence how well an insect can see in differing light conditions. As such, there is considerable variability in the number of receptor neurons that are found in the ocelli of different insects. For instance, while the ocelli of the drain fly have between two and seven photoreceptors, the number of such neurons within the fruit fly and the cabbage looper moth ocelli range from seventy to ninety [87, 88, 89]. And, at the outer extremes are the dragonfly, green bush‐cricket, and cockroach with 1500, 8000, and 10 000 photoreceptors, respectively [82, 90, 91]. Because of these differences, the ocelli actually play very diverse physiological roles in different species.

Several attributed roles for the ocelli include maintenance of stable altitude, gaze level, and orientation, in flight. This is achieved by detecting, measuring, and comparing differences in light intensity across the left and right ocellus (roll) as well as between the anterior and posterior ocellus (pitch). This is best achieved in insects that have three closely positioned ocelli [71, 76, 83, 92, 93]. In *Drosophila* and other diptera, the halteres function as gyroscopes to aid the compound eyes and ocelli in flight stabilization [94, 95, 96, 97].

The ability of the ocelli and the dorsal rim ommatidia of the compound eye to detect and distinguish polarized light from unpolarized light permits flying insects to distinguish between the ground and the sky—this allows for the identification of a sharp horizon [98]. An additional task for the ocelli is to detect small changes in light intensity over a large visual field. This is possible if the number of ocellar photoreceptors is large when compared to the number of second‐order neurons [76, 99, 100]. The most dramatic example is that of the cockroach in which the 10 000 ocellar photoreceptors converge and synapse on just four second‐order neurons [101]. Some species of desert ants combine the ability to detect polarized light, dim light from the stars, and small changes in light intensity to navigate the landscape during nightly foraging expeditions. In these instances, the ocelli function together as a celestial compass. Lastly, the ocelli are used to guide some species such as *Drosophila* toward the light [72, 75, 102, 103]. The particular type (visible or ultraviolet) and wavelength of light that the insect is attracted to will depend upon the rhodopsin gene that is expressed within ocellar photoreceptors [66, 99]. It should be noted that the compound eyes are the dominant phototactic organs and that the ocelli function to augment the phototactic response [67, 104].

All of the aforementioned behaviors require that information be very quickly transmitted from the ocelli to the brain. In general, the ocellar photoreceptors and the second‐order neurons to which they connect are much larger than their counterparts in the compound eye and its downstream circuit. Also, the number of sequential connections within the ocellar neural circuit is fewer and its wiring is much simpler than the compound eye [100, 105, 106, 107, 108, 109, 110]. As such, visual information from the ocelli is communicated to the brain at a speed several orders of magnitude faster than the data that is captured and transmitted from the compound eyes.

## Anatomy of the *Drosophila* head vertex

The dorsal head capsule (also called the head vertex) lies between the two compound eyes and is comprised of three domains—the ocellar region, the frons, and the orbital region (Fig. 1C,D) [111]. The ocellar region contains the three ocelli, two large ocellar bristles, two post vertical bristles, and six microchaetae bristles. The last set of bristles lies in between the three ocelli—this domain is called the inter‐ocellar cuticle (iOC). Immediately adjacent to the ocellar region is the frons and next to it lies the orbital region. This last domain borders the compound eye (Fig. 1D). All three domains develop from the dorsal‐anterior quadrant of the eye‐antennal disk, and each is controlled by a unique gene regulatory network.

## Early development of the head vertex

Development of the entire head vertex is dependent upon the activity of the Wingless (Wg), Hedgehog (Hh), Epidermal Growth Factor Receptor (Egfr), and Notch (N) signaling cascades as well as a suite of transcription factors. Disruptions of these pathways and their downstream transcriptional targets affect the development of various structures within the head vertex including the ocelli [112, 113, 114, 115, 116, 117]. The following is a temporal and spatial summary of the GRNs that control head vertex development within the eye‐antennal disk.

**Fig. 2:**
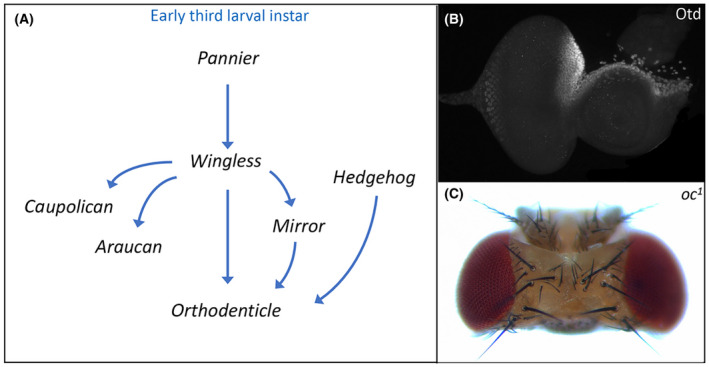
Establishment of the dorsal head vertex requires Orthodenticle (Otd). (A) Schematic of the gene regulatory network that establishes *otd* expression in the dorsal head vertex. (B) Otd protein distribution within the dorsal head vertex region of a third instar eye‐antennal disk. (C) The ocelli and frons are deleted from the adult head of *otd/oc* loss‐of‐function mutants.

A key first step in the development of the head vertex is for several of the above signaling pathways and their transcriptional targets to first activate and then refine the expression of *orthodenticle* (*otd*), also called *ocelliless* (*oc*) (Fig. 2A). Otd/Oc encodes a K_50_ class homeodomain transcription factor that is responsible for specifying the entire early ocellar field, which includes the ocelli themselves and the iOC (Fig. 2B) [118, 119]. Both structures, as well as the adjoining frons, are lost in viable *otd/oc* loss‐of‐function mutations [112, 120, 121]. As a consequence, the orbital domains expand and fuse together (Fig. 2C).

During the first larval instar, the Pannier (Pnr) transcription factor activates *wg* expression and the Wg pathway by extension throughout the entire eye‐antennal disk. The Wg pathway, in turn, stimulates *otd*/*oc* expression throughout the whole disk. As development proceeds, the Decapentaplegic (Dpp) pathway represses *wg* expression within the eye field, thereby relegating it to small domains of the dorsal and ventral margins. As a result, *otd*/*oc* expression becomes restricted to just the developing head vertex (Fig. 2B) [112, 122]. The Wg pathway also activates the expression of all three members of the Iroquois Complex—*mirror* (*mirr*), *araucan* (*ara*), and *caupolican* (*caup*) [123, 124, 125, 126]. These homeodomain transcription factors are expressed throughout the entire dorsal compartment of the eye‐antennal disk and are responsible for establishing the fate of dorsal structures including the head vertex [125, 127, 128, 129]. However, only Mirr appears to be required for ocellar development. Mirr and the Wg pathway join the Hh signaling cascade in maintaining *otd*/*oc* expression within the head vertex through the early third larval instar [112, 130]. The Wg and Hh pathways appear to directly regulate *otd/oc* as terminal transcription factors of each pathway bind to an ocellar specific enhancer [130, 131]. It is not clear if Mrr regulation of *otd/oc* is direct or indirect via intermediate factors.

**Fig. 3:**
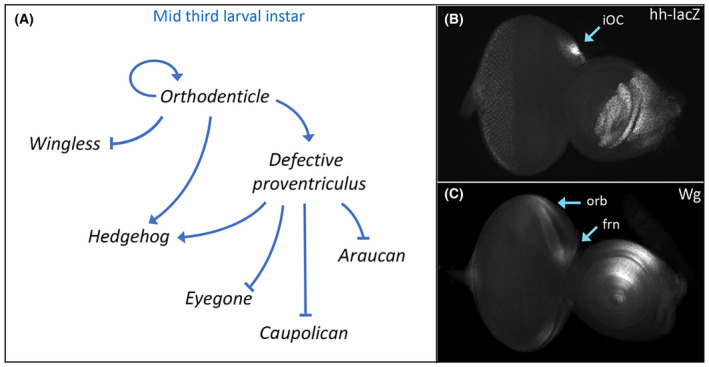
Orthodenticle regulation of *hedgehog (hh)* and *wingless (wg)* expression specifies subdomains of the head vertex. (A) Schematic of the gene regulatory network that establishes Hh signaling within the inter‐ocellar cuticle (iOC) domain and Wg signaling in the frontal bristles (frn) and orbital (orb) domain. (B) *hh‐lacZ* expression within the iOC of a third instar eye‐antennal disk. (C) Wg protein distribution within the frn and orb domains of a third instar eye‐antennal disk.

By the middle of the third larval instar, significant changes to the regulatory landscape take place with the head vertex (Fig. 3A). First, maintenance of *otd*/*oc* transcription becomes autoregulatory and independent of both Wg and Hh signaling [130]. Its expression becomes graded with high levels found within the ocellar region and ever decreasing levels within the adjacent frons and orbital regions. The differing levels of Otd/Oc appear to be important for specifying the fate of these two regions as low levels of exogenous Otd/Oc protein can rescue the frons, but higher levels are required to restore the iOC and ocelli [131]. Interestingly, overexpression of *otd*/*oc* in wild type results in the specific enlargement of the ocelli [132].

Next, the regulatory relationship between Otd/Oc, Wg, and Hh changes dramatically. Instead of being activated by Wg and Hh signaling, Otd/Oc now activates the Hh pathway and represses Wg signaling. As such, the expression patterns of these two morphogens are no longer overlapping as they were at the start of third larval instar. Instead, Hh and Wg signaling is now active in mutually exclusive domains (Fig. 3B,C). Hh signaling becomes essential for the ocellar domain while the Wg pathway specifies the adjacent frons and orbital region [112].

Lastly, within the ocellar region, Otd also the activates expression of *defective proventriculus* (*dve*) [133, 134]. Dve cooperates with Otd to maintain high levels of *hh* expression [134]. Within this same region, Dve also functions to repress transcription of the retinal determination (RD) gene *eyegone* (*eyg*) and the two IroC complex members *ara* and *caup*. Shutting off *eyg* is important for modulating the size of the ocellar region while the repression of *ara* and *caup* is essential for ensuring that the ocellar region is not forced into adopting the fates of either the orbital domain or the dorsal compound eye [134, 135].

## Development of the ocellar domain

**Fig. 4:**
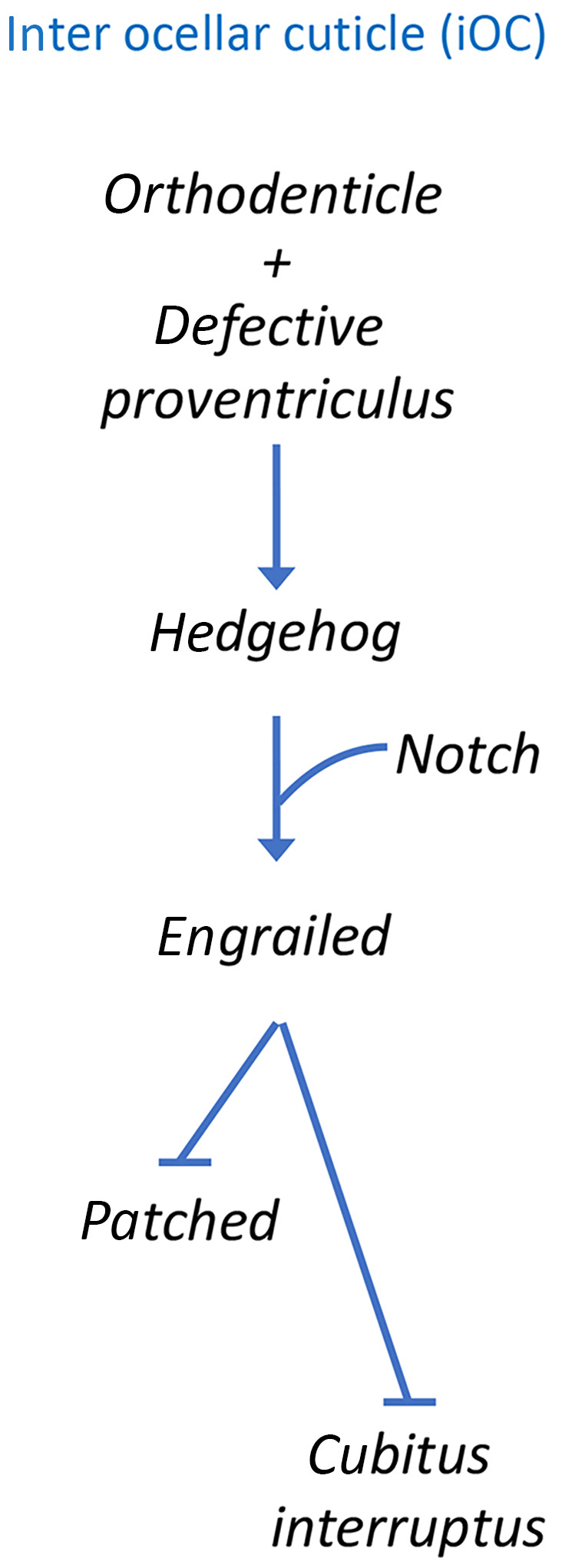
Engrailed (En) repressor specifies the fate of the iOC. Schematic of the gene regulatory network that establishes En expression within the iOC. The En repressor blocks expression of downstream target genes. This is essential for establishing the fate of the iOC. The activation of *hh* expression is important for the establishment of the neighboring ocelli. Hh signaling from the iOC non‐autonomously activates target genes in the lateral and medial ocelli.

Development of the ocellar domain can be separated into the specification of the ocelli themselves and region between the three simple eyes—the inter‐ocellar cuticle (iOC) domain. The main event within the iOC is to activate the Hh pathway, which will autonomously control development of the iOC and non‐autonomously direct formation of the medial and lateral ocelli (Fig. 4). The Hh pathway initially activates *engrailed* (*en*), whose expression is then maintained by the Notch pathway [112, 136]. En is a transcriptional repressor that is tasked with suppressing the transcription of *patched* (*ptc*) and *cubitus interruptus* (*ci*), two key members of the Hh pathway itself [137, 138, 139, 140, 141, 142]. Within the iOC, En blocks activation of the Hh pathway and this is important because the Hh pathway, if left unchecked, would transform the iOC and microchaetae bristles into ocelli. Indeed, reductions in En protein levels via loss‐of‐function mutants or disruptions to the Notch pathway eliminate the iOC. As a result, the medial and lateral ocelli are merged together to form a single large ocellus [136].

**Fig. 5:**
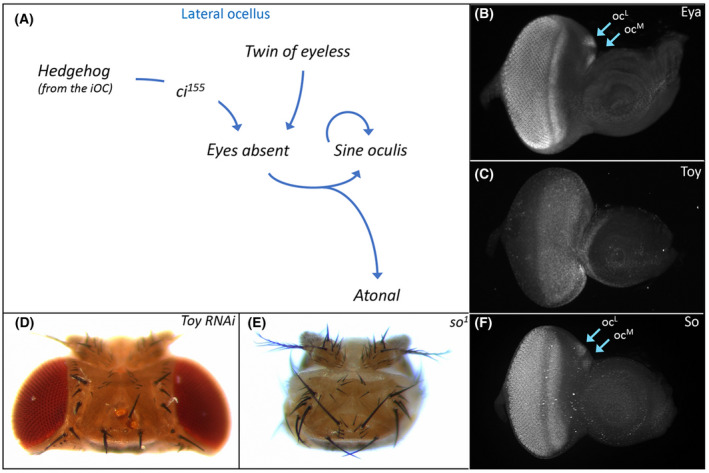
Retinal determination protein (Eya) specifies ocellar development. (A) Schematic of the gene regulatory network that establishes *eya* expression within the ocelli. Multiple inputs including both Hh signaling and Toy activate *eya* expression which in turn activates *sine oculis (so)*. The So‐Eya complex specifies the fate of the ocellus via activation of several transcription factors including Atonal (Ato). (B) Eya protein distribution within the compound eye and both ocelli of a third instar eye‐antennal disk. (C) Toy protein distribution within the dorsal head vertex domain and adjacent head epidermal region of the antenna of a third instar eye‐antennal disk. (D) Variable numbers of ocelli are lost from the adult head when *toy* expression is knocked down with RNAi. (E) Sine Oculis (So) protein distribution within the compound eye and both ocelli of a third instar eye‐antennal disk. (F) The compound eyes and ocelli are completely lost from the adult head in *so* loss‐of‐function mutants.

Hh signaling within the iOC influences the development of the adjacent ocelli via two non‐autonomous signaling mechanisms. First, Hh signaling effects ocellar development by non‐autonomously activating expression of a portion of the RD network within the ocelli (Fig. 5A). One such target is *eyes absent* (*eya*), which is expressed throughout the compound eyes and ocelli and encodes a transcription factor with both transcriptional activator and tyrosine phosphatase activity (Fig. 5B) [143, 144, 145, 146, 147]. Two viable, loss‐of‐function mutant alleles of *eya* exist (*eya^1^*, *eya^2^*). In both strains, the compound eyes are completely missing but the ocelli appear normal in appearance [148, 149]. A molecular analysis of *eya^1^* and *eya^2^* determined that the ocelli remain because an enhancer element that drives expression in the developing eye but not the ocelli is deleted in both mutant alleles [150]. A search for additional regulatory elements identified an enhancer that drives expression within the ocelli. And, as expected, the removal of this element eliminates the ocelli [151]. This is consistent with the loss of ocelli that is seen when an *otd/oc* enhancer is used to drive expression of an *eya* RNAi construct [152]. Hh signaling from the iOC activates transcription of *eya* in both the medial and lateral ocelli [130]. It remains an open question if the Hh pathway, via the Cubitus interruptus (Ci) transcription factor, directly binds to and activates the ocellar enhancer.

A second input into *eya* appears to be the Pax6 transcription factor Twin of Eyeless (Toy). Toy occupies the highest genetic position within RD network and is expressed throughout the developing eye and ocellar regions from the earliest stages of development (Fig. 5C) [153]. Toy as well as its paralog and downstream target Eyeless (Ey) are required for the formation of the entire eye‐antennal disk. When expression of both genes is simultaneously knocked down (to eliminate all Pax6 function), the eye‐antennal disks fail to form and the resulting pharate adults are headless [154]. Similarly, the vast majority of *toy^hdl^* and *toy^1^* null mutants also lack the eye‐antennal disks [154, 155]. However, a small number of both mutant alleles do survive to adulthood and have ocellar defects [132, 152, 155, 156]. This is consistent with the loss of ocelli that is seen when *toy* is knocked down via RNAi just within the ocellar domain (Fig. 5D) [152]. *eya* is thought to be regulated by Toy because its expression within the ocelli is severely disrupted in *toy* mutants while the overexpression of *toy* has the opposite effect [132]. It is not clear, however, if Toy binds to the ocellar specific enhancer of *eya* that was identified in [151] and activates its expression. In the compound eye, Ey and Toy do not appear to directly activate *eya* expression.

In the compound eye, Eya forms a biochemical complex with the homeobox transcription factor Sine Oculis (So) [157]. The So‐Eya complex is an integral part of the RD network and functions to both promote an eye fate and to suppress the formation of head epidermis [143, 157, 158, 159, 160, 161]. The first evidence that *so* is important for ocellar development came from the viable *so^1^* mutant—adult flies lack both the compound eyes and the ocelli (Fig. 5E) [162]. As expected, *so* is expressed in both visual systems (Fig. 5F). As with the viable *eya^1^* and *eya^2^* alleles, s*o^1^* flies harbor a spontaneous deletion of an enhancer element [158]. In this instance, the deletion is large enough to encompass separate eye and ocellar regulatory sequences [163, 164, 165]. While *so* expression is directly activated by both Toy and Ey in the compound eye [164, 166], its initial expression in the ocelli appears to be dependent upon Eya [132]. Afterward, maintenance of *so* expression within each ocellus is controlled by an autoregulatory loop that is independent of both Pax6 proteins [132, 165].

Within the ocelli, the So‐Eya complex goes on to activate the expression of the proneural gene *atonal (ato)* via control of an enhancer element (Fig. 5A) [167]. The complex similarly regulates *ato* expression in the developing eye as well [168]. In the compound eye, *ato* is expressed in and required for the formation of the R8 photoreceptor [169, 170, 171]. This in turn triggers the stepwise recruitment of the remaining photoreceptor neurons, which can be divided into several additional subtypes R2/5, R3/4, R1/6, and R7 [2, 16, 169, 172, 173]. Within a developing ocellus only a subset of the approximately 80 photoreceptor neurons express *ato*. This could suggest that although an R7‐like cell may not exist (see above) other neuronal subtypes might exist in the ocellus as they do in the ommatidium of the compound eye.

In addition to *ato*, a number of other targets of the So‐Eya complex have been identified in the developing eye [174, 175, 176, 177, 178, 179, 180]. A few of these targets, including *ey,* are not expressed within the developing ocelli [181] and thus represent examples of how the gene regulatory networks that underlie the two visual systems differ from each other. In contrast, several targets such as *glass* (*gl*), *pointed* (*pnt*), and the RD network gene *dachshund* (*dac*) are expressed in both types of eyes [175, 182, 183, 184, 185]. The *gl* gene is one of the best studied So‐Eya targets. Loss‐of‐function mutations that disrupt *gl* eliminate photoreceptor formation in both the compound eyes and ocelli [186]. An enhancer that drives expression within the ocelli has been recently identified [185], and it will be interesting to determine whether its activation is directly dependent upon the So‐Eya complex. We note here that *gl* is also expressed in adult ocelli and is required for photoreceptor maintenance and proper ocellar function [28, 30].

**Fig. 6:**
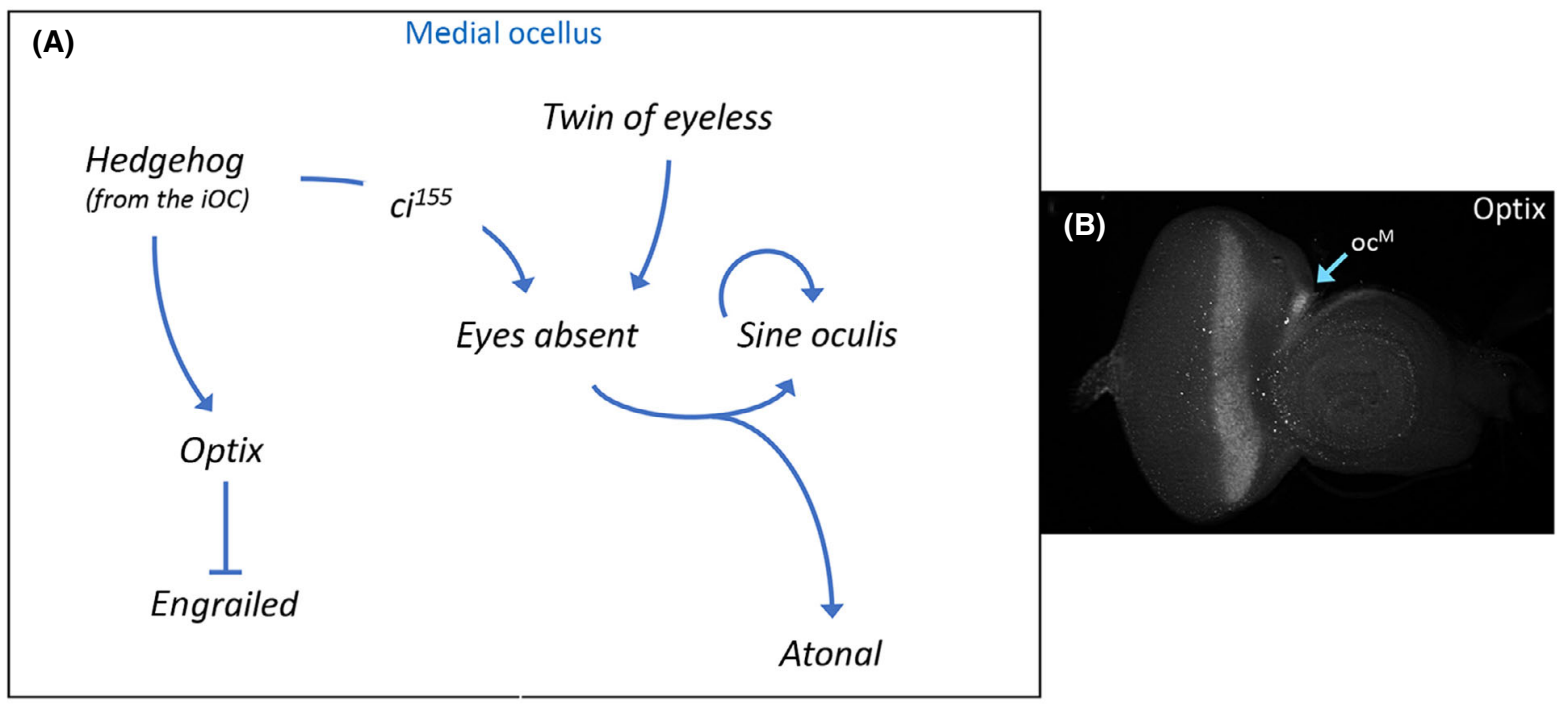
Retinal determination protein Optix prevents the medial ocellus from adopting the fate of iOC. (A) Schematic of the gene regulatory network that describes how the fate of the ocelli is established. The So‐Eya complex specifies the fate of the ocelli by activating photoreceptor‐specific genes while other factors simultaneously preventing the ocellus from adopting the fate of the iOC (via repression of *en*). In the medial ocellus, the repression of *en* is mediated by the retinal determination protein Optix. Other factor(s) are likely to play a similar role in repressing *en* expression within the lateral ocellus. (B) Optix protein is found just within just the medial ocellus of a third instar eye‐antennal disk.

Hh signaling from the iOC also activates expression of the RD network member *optix* just within the medial ocellus (Fig. 6A,B) [187]. Optix is the *Drosophila* homolog of vertebrate Six3/6 and encodes a transcriptional repressor [188, 189, 190]. In addition to its role in specifying the fate of the compound eye, *optix* is also required for the progression of the morphogenetic furrow [189, 191]. Its role in ocellar development is to inhibit the expression of *en* within the medial ocellus (Fig. 6A) [187]. This allows for Hh signaling to be activated in the medial ocellus, which is essential for the downstream GRN (described above) to be activated. The medial ocellus is lost when *optix* expression is knocked down. Its loss is caused by the upregulation of En expression within the medial ocellus [187]. As *optix* is only expressed in the medial ocellus, other factors must be present in the lateral ocellus to further restrict *en* expression.

**Fig. 7:**
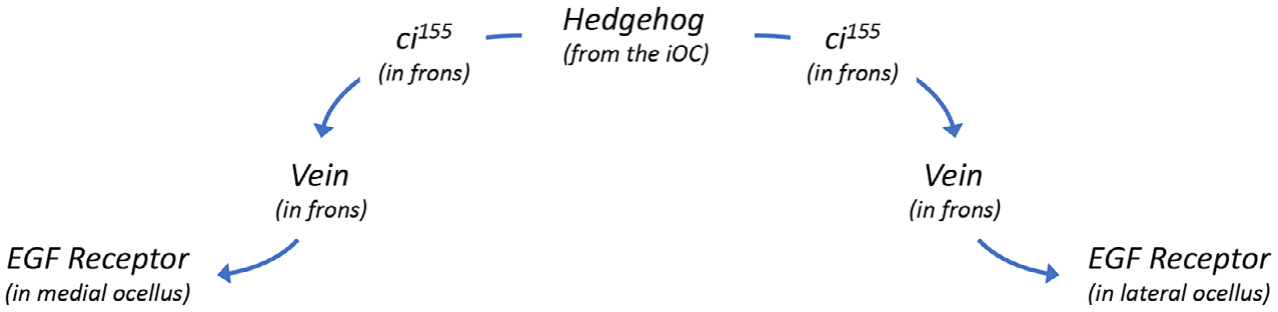
Relay system involving Hedgehog and EGF Receptor (EGFR) signaling controls ocellar development. Schematic diagram of a non‐autonomous signaling system that controls ocellar development. Previous figures described how Hh signaling from the iOC is received within each ocellus. In this figure, we describe a second Hh‐dependent system. Hh emanating from the iOC activates expression of the EGFR ligand *vein (vn)* within the frons. Vn from the frons then activates EGFR signaling within the both ocelli via the Pointed (Pnt) transcription factor.

The other mechanism by which Hh signaling from the iOC controls ocellar development involves complex signaling through the neighboring frons (Fig. 7). Hh signaling from the iOC activates expression of *vein* (*vn*), a ligand for the EGF Receptor, within the adjoining frons [116]. Vn then signals back and activates EGFR signaling within the ocelli themselves. The most downstream transcription factor of the EGFR pathway, *pointed* (*pnt*), is expressed within the ocelli [192]. Loss‐of‐function EGFR alleles, expression of a dominant‐negative EGFR protein, and knockdown of *pnt* via RNAi all result in the elimination of the ocelli and associated bristles [114, 192, 193]. EGF Receptor signaling works with Otd and Dve to maintain *otd* expression within the ocellar region [194].

## Conclusions

The vast array of genetic and molecular tools that are available to *Drosophila* researchers has established the fly as a premier system to study important topics in developmental biology such as fate specification and tissue patterning [195]. For over a century, these tools have been applied to the study of the compound eye in several thousand individual studies. So, while our knowledge of the compound eye is far from complete, it is both vast and deep. By comparison, our understanding of how the ocellar visual system develops is still in its infancy even though the ocelli control a broad array of essential behaviors and share portion of the same gene regulatory network as the compound eye. Moreover, since the compound eyes and ocelli are likely to have arisen from a common ancestral visual system, studies of the ocelli will put us in a strong position to understand how these two organs have evolved [196]. It is our hope that this Viewpoint article renews interest in ocellar visual system development.

## Conflict of interest

The authors declare no conflict of interest.

## Author contributions

CBJG wrote and edited the manuscript. JPK wrote and edited the manuscript and secured funding for the project.

## Data Availability

All data within this manuscript is available immediately upon request.
